# Influence of Androgen Receptor Gene CAG and GGC Polymorphisms on Male Sexual Function: A Cross-Sectional Study

**DOI:** 10.1155/2016/5083569

**Published:** 2016-12-15

**Authors:** Giacomo Tirabassi, Giovanni Corona, Sara Falzetti, Nicola delli Muti, Mario Maggi, Giancarlo Balercia

**Affiliations:** ^1^Division of Endocrinology, Department of Clinical and Molecular Sciences, Umberto I Hospital, Polytechnic University of Marche, Via Conca 71, 60126 Ancona, Italy; ^2^Endocrinology Unit, Medical Department, Maggiore-Bellaria Hospital, Azienda-Usl Bologna, Bologna, Italy; ^3^Sexual Medicine and Andrology Unit, Department of Experimental, Clinical and Biomedical Sciences, University of Florence, Florence, Italy

## Abstract

*Background*. No study has assessed the possible involvement of GGC androgen receptor (AR) polymorphism in sexual function. Our aim is to evaluate the association between CAG and GGC AR polymorphisms in this function. *Methods*. We retrospectively examined eighty-five outpatients. Clinical, biochemical, and genetic parameters were considered. Sexual assessment was performed using the International Index of Erectile Function (IIEF) which evaluates erectile function (EF), orgasmic function (OF), sexual desire (SD), intercourse satisfaction (IS), and overall satisfaction (OS). *Results*. In the whole sample, CAG repeats were inversely correlated with EF, OF, and total IIEF-15 score, whereas GGC tracts did not show any significant correlation with sexual function. CAG relationship with IIEF items retained significance only in the eugonadal but not in the hypogonadal cohort. On the other hand, GGC tracts were not found to be significantly correlated with IIEF variables in either eugonadal or hypogonadal subjects. In eugonadal subjects, logistic regression pointed out that a higher number of CAG triplets were associated with lower values of EF, OF, SD, OS, and total IIEF independently from other confounders. *Conclusions*. GGC polymorphism seems not to exert any influence on sexual function, whereas CAG polymorphism appears to affect sexual parameters only in eugonadal subjects.

## 1. Introduction

Testosterone, along with its metabolite dihydrotestosterone, exerts its effects by binding to the androgen receptor (AR) [1–3]. CAG and GGC trinucleotide repeats are two polymorphic segments located in the amino-terminal transactivation domain of the AR gene and are able to affect the peripheral effects of testosterone [4, 5]. Polymorphic sequences of CAG repeats may range in number from 10 to 35 and encode polyglutamine stretches of the AR transactivation domain [6, 7]. Although controversial findings have been reported, literature generally shows that CAG number is negatively correlated to the transcriptional activity of AR and, as a result, to the effects of testosterone [6, 8, 9]. On the other hand, in the AR protein, a polymorphic polyglycine tract is present and it is encoded by an invariant six-glycine tract (GGT/GGG) followed by a polymorphic GGC repeat [4], which varies from 10 to 35. Of note, most authors refer to the polyglycine tract as being encoded by a GGN repeat, which is equivalent to the GGC repeat plus six triplets [10]. The median number of GGN repeats is 23 in normal men [11]. The polyglycine tract is located in the transactivating domain of the AR protein, suggesting an effect of repeat length on receptor function. The functional consequences of variations in the GGC repeat have not yet been completely understood. However, an in vitro study indicated that the AR protein is inversely regulated by glycine repeat length, thus resulting in an increased amount of AR activity in cells expressing AR with a shorter glycine repeat [4].

Many works have assessed the relationship between androgen receptor CAG and GGC polymorphisms and clinical parameters related to testosterone activity (i.e., metabolic profile, bone density, and prostate cancer) [6, 12–14]. However, little information is present about the relationship between the polymorphisms and sexual function. In fact, even if the relationship between CAG repeat and sexual function has been evaluated by some cross-sectional studies reporting quite contradictory results [15–19], no study has assessed the possible influence of GGC polymorphism on that parameter so far.

Therefore, the aim of the present study is to perform an evaluation of the possible association between the two AR polymorphisms and sexual function in a cohort of patients consulting for sexual dysfunction.

## 2. Materials and Methods

### 2.1. Subjects

This retrospective study evaluated outpatients consulting at our andrological unit between 2010 and 2016 for sexual dysfunctions. Selection of subjects was made on the basis of patient characteristics present at the moment of their first evaluation. Inclusion criteria were (1) absence of important systemic diseases, both previous and ongoing, apart from late-onset hypogonadism or type 2 diabetes mellitus; (2) availability of all the considered clinical, biochemical, and genetic parameters; (3) sexual relationship since at least 1 year before the visit; and (4) absence of previous investigation or treatment for sexual dysfunction. Exclusion criteria were (1) ongoing pharmacological treatment for hypogonadism; (2) presence of endocrinological disorders other than late-onset hypogonadism or type 2 diabetes mellitus; (3) alcohol or drug dependence; and (4) psychiatric diseases.

Late-onset hypogonadism was defined according to previous accepted biochemical criteria (fasting levels of serum total testosterone <2.31 ng/mL or, in the case of total testosterone between 2.31 and 3.46 ng/mL, calculated free testosterone <65 pg/mL) together with signs and symptoms consistent with hypogonadism [20, 21].

Eighty-five patients were finally eligible for this retrospective study.

In order to determine hypogonadism or eugonadism, at least two measurements of testosterone were performed.

All the clinical and biochemical parameters were performed in the context of the clinical work-up. On the contrary, genetic analyses were carried out in the context of previous research protocols. Proper communication of this retrospective analysis was given to our institutional review board, which approved it.

### 2.2. Clinical Parameters

The following clinical parameters were considered: age and body mass index (BMI). BMI was calculated according to standard methods previously described [22]. Presence of type 2 diabetes mellitus, dyslipidemia, hypertension, and smoking habit was also considered. Specifically, smoking habit was defined as having smoked at least 100 cigarettes in a lifetime [23, 24].

### 2.3. Biochemical Parameters

The following biochemical parameters were considered: follicle-stimulating hormone (FSH), luteinizing hormone (LH), total testosterone, calculated free testosterone, sexual hormone-binding globulin (SHBG), and estradiol. Blood samples for FSH, LH, total testosterone, SHBG, and estradiol were taken in the morning after overnight fast, and their assay was carried out as previously specified [12].

The normal reference ranges for the biochemical parameters studied were the following: FSH, 1.7–6.9 IU/L; LH, 1.6–10.0 IU/L; total testosterone, 3–8.5 ng/mL; SHBG, 20–60 nmol/L; and estradiol, 11–47 pg/mL.

Free testosterone was calculated according to Vermeulen's formula (at http://www.issam.ch/freetesto.htm) [25].

### 2.4. Sexual Assessment

Sexual assessment was evaluated using the International Index of Erectile Function-15 (IIEF-15) which provides a score for erectile function (EF), orgasmic function (OF), sexual desire (SD), intercourse satisfaction (IS), and overall satisfaction (OS) [26, 27]. The IIEF domains for EF (items 1, 2, 3, 4, 5, and 15), OF (items 9 and 10), SD (items 11 and 12), IS (items 6, 7, and 8), and OS (items 13 and 14) were used to evaluate sexual function. According to the EF score, EF can be normal (26–30), slightly impaired (17–25), moderately impaired (11–16), or severely impaired (less than 11). Regarding OF (score range 0–10), SD (score range 2–10), IS (score range 0–15), and OS (score range 2–10), the higher the score the better the parameter studied. Total IIEF-15 score was calculated.

### 2.5. CAG and GGC AR Polymorphism Assessment

Genetic parameters were evaluated as previously described [12]. Briefly, genomic DNA was extracted from whole blood, using the QIAamp DNA Blood Mini Kit (Qiagen, Hilden, Germany). CAG and GGC repeats were amplified through PCR using appropriate primers [28], and PCR products were purified using a PCR purification kit. Sequencing reaction was performed with the BigDye Terminator Cycle Sequencing Ready Reaction Kit (ABI Prism, Applied Biosystems), and the samples obtained were sequenced on a CEQ 2000 XL sequencer (Beckman Coulter, Fullerton, CA, USA).

### 2.6. Statistical Analysis

Shapiro-Wilk's test was applied to verify the normal distribution of the continuous variables. Continuous variables are presented as mean and standard deviation if normally distributed or as median and interquartile range if not normally distributed. Comparisons between the two groups were performed with Mann–Whitney's *U* test because of the skewed distribution of data. Fisher's exact test was adopted for comparisons between the frequencies of the two groups. Spearman correlation was performed to analyze the relationships of clinical, hormonal, and genetic variables with sexual parameters. Continuous variables which were significantly correlated with sexual parameters at bivariate correlations or categorical clinical variables (i.e., presence/absence of type 2 diabetes mellitus, dyslipidemia, hypertension, and smoking habit) which differed in sexual profile were entered into logistic regression models in order to assess their independent association with sexual parameters. In order to fit with logistic regression, sexual parameters (dependent variables) were dichotomized according to their median value. Dependent variables are categorized as 0 (low values) and 1 (high values).

Significance was set at *p* < 0.05. Statistical analyses were performed using SPSS 16 package (SPSS Inc., Chicago, IL, USA).

## 3. Results

Table 1 shows the clinical, hormonal, and genetic characteristics of the studied subjects. After categorizing the sample according to gonadal status, hypogonadal subjects were found to be older and with a higher prevalence of diabetes mellitus when compared to the eugonadal ones. In addition, they showed worse total and subdomain IIEF scores (Table 1).

In the whole sample, age was negatively correlated with all sexual function variables, whereas total testosterone and estradiol levels showed positive correlations with all sexual parameters (Table 2). Of note, CAG repeats were inversely correlated with EF, OF, and total IIEF-15 score, whereas GGC tracts did not show any significant correlations with sexual function (Table 2).

We subsequently evaluated the same correlations categorizing the cohort according to gonadal status (Table 3). In hypogonadal patients, age was negatively correlated with sexual function, whereas total testosterone was positively correlated with EF, OF, SD, and total IIEF score. Neither CAG nor GGC repeats correlated significantly with sexual parameters (Table 3). On the other hand, in eugonadal subjects, age was negatively correlated with all sexual parameters, apart from SD. In addition, in eugonadal subjects, CAG repeats were negatively correlated with all IIEF parameters, but GGC tracts were not found to be significantly correlated with any of the assessed variables (Table 3).

Among the considered clinical comorbidities, in the whole sample, only diabetes mellitus influenced sexual function as diabetic subjects showed a worse sexual function than nondiabetic ones (Table 4). On the other hand, hypogonadal subjects with dyslipidemia and smoking habit had worse sexual parameters than nonaffected patients and nonsmokers, thus suggesting an effect on sexual function of these conditions (Table 4). Conversely, in eugonadal subjects, only hypertension was able to affect sexual function as hypertensive subjects had worse sexual function than normotensive ones (Table 4).

In eugonadal subjects, logistic regression models were performed in order to establish the independent contribution of CAG repeat polymorphism on sexual function (Table 5). After adjustment for age and presence of hypertension, a higher number of CAG triplets were associated with low values of EF, OF, SD, OS, and total IIEF. In addition, higher age was associated with lower values of OF and OS. Finally, presence of hypertension was associated with lower values of EF, OF, IS, OS, and total IIEF.

In hypogonadal subjects, logistic regression was not performed since, at bivariate correlations, the genetic parameters were not found to be significantly correlated with any of the sexual parameters.

## 4. Discussion

This study confirms the view [16] that, in eugonadal subjects, a longer length of the polymorphic CAG repeats in AR can impair several androgen-dependent functions, such as sexual function. However, the most important and novel finding of this work is reporting a lack of association between GGC repeat polymorphism and sexual function, in both hypogonadal and eugonadal subjects. This is the first study examining a possible direct association between GGC and sexual dysfunctions, as captured by IIEF. In fact, GGC polymorphism has not been evaluated as carefully as CAG. GGC polymorphism could influence sexual function by directly conditioning AR activity or by modulating the patient metabolic profile. Accordingly, growing evidence suggests a role for GGC polymorphism in regulating several metabolic pathways of men [10, 12, 29]. More specifically, we have recently found that, in men, GGC triplets are positively and independently associated with glycemia, glycated hemoglobin, total cholesterol, triglycerides, and insulin resistance (homeostasis model), thus suggesting a relevant role of GGC repeat tracts in stratifying male cardiovascular risk [12]. However, the present work ruled out the confounding role of the metabolic status in the relationship between sexual function and GGC polymorphism.

On the other hand, we found that longer CAG polymorphism was significantly associated with worse sexual parameters, although this finding was evident only in eugonadal subjects. Our results are in line with those reported by Liu et al. [16] who recently showed that, after adjusting for other covariates, a longer AR CAG repeat length was an independent risk factor for erectile dysfunction only in subjects with total testosterone above 3.3 ng/mL. The same group carried out a free health screening in men older than 40 years, and they found that when total testosterone levels were above 3.40 ng/mL, subjects with AR CAG repeat lengths >25 had a significantly higher risk of developing testosterone deficiency symptoms (ADAM questionnaire) than those with AR CAG repeat lengths <22. Interestingly, this was not observed when total testosterone levels were equal to or less than 3.40 ng/mL [17]. Similarly, Pastuszak et al. [15], by reviewing the medical records of 85 men who presented to their clinic, found that AR gene CAG repeat number was negatively related to all domains of sexual function as assessed by IIEF-15. Conversely, another study, conducted on 213 men aged 41–70 years randomly selected from the population registry of a Finnish city, indicated that men with CAG repeats higher than or equal to 23 reported decreased potency (assessed by the Heinemann questionnaire) less often than the others [18]. In addition, Andersen et al. [19] evaluated 79 men with erectile dysfunction and 340 controls in a population-based survey, and they found no significant association between erectile dysfunction symptomatology and CAG repeat length. However, those authors, when evaluating erectile dysfunction complaints, used just a single question taken from the National Institutes of Health Consensus Development Panel on Impotence (1993) [19].

Only two reports have evaluated the effects of CAG polymorphism on conditioning sexual function recovery after testosterone therapy in hypogonadal male patients. They both found that shorter CAG length is associated with a greater improvement of several aspects of the IIEF questionnaire [7, 30]. However, it must be emphasized that one of these two reports evaluated a very limited number of subjects (*n* = 15) with hypogonadotropic hypogonadism, a very rare form of male hypogonadism [7]. Moreover, in the same study, subjects were undergoing pituitary replacement therapy and results were obtained after statistical adjustment for those confounding factors [7].

The findings of the present work support the model of androgen effects conceived by Zitzmann [31]. In fact, this author previously hypothesized that genetically determined functional differences in AR activity can be observed only within the range of eugonadal testosterone levels, as the receptor needs a normal amount of substrate (testosterone) to exhibit the effects of polymorphism [31]. Conversely, in hypogonadism, testosterone-related effects will be strongly dependent on androgen levels, as testosterone binds to AR and will increase androgen effects until saturation of the receptors is reached [31].

Before concluding, it should be acknowledged that because the study population is retrospectively enrolled from an outpatient clinic and the patient number is relatively small, a selection bias could exist and the results may not be extended to the general population. This is a study limitation and further large epidemiologic studies will be needed to confirm our preliminary findings.

In conclusion, our work has ruled out the influence of GGC androgen receptor polymorphism on sexual function regulation in both hypogonadal and eugonadal subjects. On the other hand, we confirmed the role of CAG polymorphism in influencing sexual parameters in eugonadal subjects but not in hypogonadal ones. However, future research should explore the hypothesized possible influence of cofactors [12, 32] on the relationship between AR polymorphisms and testosterone-related clinical features.

## Figures and Tables

**Table 1:** Clinical, hormonal, and genetic characteristics of the studied subjects.

	Total sample (*n* = 85)	Hypogonadal subjects (*n* = 43)	Eugonadal subjects (*n* = 42)	*p* ^*∗*^
Age (years)	64 (59.5–69.5)	69 (68–73)	59.5 (57.7–61.2)	<0.001
BMI (kg/m^2^)	28 (25.5–30.9)	27.8 (25.9–34.9)	28.4 (25–29.9)	NS
Type 2 diabetes mellitus (no/yes) (%)	39/46 (54.1)	12/31 (72.1)	27/15 (35.7)	0.001
Dyslipidemia (no/yes) (%)	47/38 (44.7)	23/20 (46.5)	24/18 (42.9)	NS
Hypertension (no/yes) (%)	48/37 (43.5)	23/20 (46.5)	25/17 (40.5)	NS
Smoking habit (no/yes) (%)	31/54 (63.5)	14/29 (67.4)	17/25 (59.5)	NS
FSH (IU/L)	12.5 (7.3–17.2)	17.2 (12.9–22.9)	8.2 (3.4–11.8)	<0.001
LH (IU/L)	10.8 (4–15.6)	15.6 (12.8–19.1)	4 (2.7–7.3)	<0.001
Total testosterone (ng/mL)	2.97 (2.73–5.12)	2.75 (2.28–2.88)	5.1 (4.3–6)	<0.001
Free testosterone (pg/mL)	63.8 (52.7–119)	52.8 (45–57.9)	119 (96.3–138.2)	<0.001
SHBG (nmol/L)	30.6 ± 3.48	32.1 (28.6–34.5)	30.4 (28.1–32.2)	NS
Estradiol (pg/mL)	16.5 (7.7–31.8)	7.8 (5.6–11.5)	31.8 (27.2–37.1)	<0.001
CAG repeats	18 (14–21)	19 (14–21)	17 (14–19.2)	NS
GGC repeats	19 (17–21)	18 (16–22)	20 (18–21)	NS
EF	17 (10–22)	10 (8–13)	22 (21–24)	<0.001
OF	7 (5–9)	5 (4–6)	9 (8‐9)	<0.001
SD	7 (4–8)	4 (4–6)	8 (8‐8)	<0.001
IS	8 (6–13)	6 (6‐7)	13 (12.7–13)	<0.001
OS	4 (3–9)	3 (3‐4)	9 (8‐9)	<0.001
Total IIEF-15 score	43 (29–61)	29 (25–36)	61 (57.7–63)	<0.001

Continuous variables are presented as mean ± standard deviation if normally distributed or as median (interquartile range) if not normally distributed.

^*∗*^Comparison between hypogonadal and eugonadal subjects.

BMI = body mass index; SHBG = sexual hormone binding globulin; EF = erectile function; OF = orgasmic function; SD = sexual desire; IS = intercourse satisfaction; OS = overall satisfaction; IIEF = International Index of Erectile Function; NS, not significant.

**Table 2:** Correlations of clinical, hormonal, and genetic variables with sexual parameters in the whole sample.

	EF	OF	SD	IS	OS	Total IIEF-15 score
Age	*r*: −0.923 *p* < 0.001	*r*: −0.922 *p* < 0.001	*r*: −0.911 *p* < 0.001	*r*: −0.901 *p* < 0.001	*r*: −0.898 *p* < 0.001	*r*: −0.926 *p* < 0.001
BMI	NS	NS	NS	NS	NS	NS
Total testosterone	*r*: 0.808 *p* < 0.001	*r*: 0.807 *p* < 0.001	*r*: 0.818 *p* < 0.001	*r*: 0.798 *p* < 0.001	*r*: 0.790 *p* < 0.001	*r*: 0.806 *p* < 0.001
Estradiol	*r*: 0.777 *p* < 0.001	*r*: 0.777 *p* < 0.001	*r*: 0.804 *p* < 0.001	*r*: 0.771 *p* < 0.001	*r*: 0.766 *p* < 0.001	*r*: 0.772 *p* < 0.001
CAG repeats	*r*: −0.228 *p*: 0.036	*r*: −0.231 *p*: 0.033	NS	NS	NS	*r*: −0.226 *p*: 0.037
GGC repeats	NS	NS	NS	NS	NS	NS

BMI = body mass index; EF = erectile function; OF = orgasmic function; SD = sexual desire; IS = intercourse satisfaction; OS = overall satisfaction; IIEF = International Index of Erectile Function; NS, not significant.

**Table 3:** Correlations of clinical, hormonal, and genetic variables with sexual parameters in hypogonadal and eugonadal subjects.

	Hypogonadal subjects						Eugonadal subjects					
	EF	OF	SD	IS	OS	Total IIEF-15 score	EF	OF	SD	IS	OS	Total IIEF-15 score
Age	*r*: −0.914 *p* < 0.001	*r*: −0.942 *p* < 0.001	*r*: −0.892 *p* < 0.001	*r*: −0.699 *p* < 0.001	*r*: −0.761 *p* < 0.001	*r*: −0.923 *p* < 0.001	*r*: −0.452 *p*: 0.003	*r*: −0.415 *p*: 0.006	NS	*r*: −0.461 *p*: 0.002	*r*: −0.414 *p*: 0.006	*r*: −0.474 *p*: 0.002
BMI	NS	NS	NS	NS	NS	NS	NS	NS	NS	NS	NS	NS
Total testosterone	*r*: 0.480 *p*: 0.001	*r*: 0.416 *p*: 0.006	*r*: 0.389 *p*: 0.010	NS	NS	*r*: 0.476 *p*: 0.001	NS	NS	NS	NS	NS	NS
Estradiol	NS	NS	NS	NS	NS	NS	NS	NS	NS	NS	NS	NS
CAG repeats	NS	NS	NS	NS	NS	NS	*r*: −0.624 *p* < 0.001	*r*: −0.508 *p*: 0.001	*r*: −0.510 *p*: 0.001	*r*: −0.588 *p* < 0.001	*r*: −0.508 *p*: 0.001	*r*: −0.618 *p* < 0.001
GGC repeats	NS	NS	NS	NS	NS	NS	NS	NS	NS	NS	NS	NS

BMI = body mass index; EF = erectile function; OF = orgasmic function; SD = sexual desire; IS = intercourse satisfaction; OS = overall satisfaction; IIEF = International Index of Erectile Function; NS, not significant.

**Table 4:** Comparison of sexual function between subjects affected and not affected by type 2 diabetes mellitus, dyslipidemia, and hypertension and with or without smoking habit.

	Type 2 diabetes mellitus		Dyslipidemia		Hypertension		Smoking habit	
	Yes	No	Yes	No	Yes	No	Yes	No
Whole sample								
EF	13 (8–21)	21 (14–23)^*∗*^	16 (8–22)	21 (11–23)	17 (7.5–21)	20 (10–23)	14 (9–22)	21 (13–23)
OF	6 (4–8)	8 (6–9)^*∗*^	7 (4–9)	8 (6–9)	7 (4–8)	7.5 (5–9)	6 (4–9)	8 (6–9)
SD	5 (4–8)	8 (6–8)^*∗*^	7 (4–8)	8 (5–8)	7 (4–8)	8 (4–8)	6 (4–8)	8 (5–8)
IS	7 (6–12.2)	13 (7–13)^*∗*^	7 (6–13)	12 (7–13)	8 (6–12)	11 (7–13)	7 (6–13)	12 (7–13)
OS	4 (3–8)	8 (4–9)^*∗*^	4 (3–9)	8 (3–9)	4 (3–8)	6.5 (3–9)	4 (3–9)	8 (4–9)
Total IIEF-15 score	35 (25–57.2)	58 (36–62)^*∗*^	41 (25–61)	57 (32–62)	43 (24.5–57)	53 (29–62)	37 (26–61)	57 (35–62)

Hypogonadal subjects								
EF	10 (7–13)	11 (9–13.7)	8 (7–10.7)	11 (10–14)^*∗*^	8 (7–15.7)	10 (9–11)	9 (7.5–11)	12.5 (9.5–16)^*∗*^
OF	5 (4–6)	5.5 (4–6)	4 (4–5.7)	6 (4–6)^*∗*^	4 (4–7)	5 (4–6)	4 (4–6)	6 (4.7–7)^*∗*^
SD	4 (4‐5)	4.5 (4–6)	4 (4–4.7)	5 (4–6)^*∗*^	4 (4–6.7)	4 (4‐5)	4 (4‐5)	5 (4–7)^*∗*^
IS	6 (6‐7)	6.5 (6‐7)	6 (6–6.7)	7 (6‐7)^*∗*^	6 (6‐7)	7 (6‐7)	6 (6‐7)	7 (6‐7)
OS	3 (3‐4)	3 (3‐4)	3 (3‐3)	3 (3‐4)^*∗*^	3 (3‐4)	3 (3‐3)	3 (3‐3)	4 (3‐4)^*∗*^
Total IIEF-15 score	29 (24–35)	30.5 (26.2–36)	25 (24–30.5)	32 (28–37)^*∗*^	25 (24–40.5)	29 (26–32)	27 (24.5–31.5)	34 (28–41)^*∗*^

Eugonadal subjects								
EF	22 (21–25)	22 (21–23)	22 (21–23.2)	22.5 (21–25)	21 (21‐21)	23 (22–25)^*∗*^	22 (21–24)	23 (21–24)
OF	9 (8‐9)	9 (8‐9)	9 (8‐9)	9 (8‐9)	8 (8‐8)	9 (9‐9)^*∗*^	9 (8‐9)	9 (8‐9)
SD	8 (8‐8)	8 (8‐8)	8 (8‐8)	8 (8‐8)	8 (8‐8)	8 (8‐8)	8 (8‐8)	8 (8‐8)
IS	13 (12–14)	13 (13‐13)	13 (12‐13)	13 (13‐14)	12 (12‐13)	13 (13‐14)^*∗*^	13 (12–13.5)	13 (13‐13)
OS	9 (8‐9)	9 (8‐9)	9 (8‐9)	9 (8‐9)	8 (8‐8)	9 (9‐9)^*∗*^	9 (8‐9)	9 (8‐9)
Total IIEF-15 score	61 (57–65)	61 (58–62)	61 (57–62.2)	61.5 (58–65)	57 (57‐58)	62 (61–65)^*∗*^	61 (57–63.5)	62 (58–63)

Data are presented as median and interquartile range. EF = erectile function; OF = orgasmic function; SD = sexual desire; IS = intercourse satisfaction; OS = overall satisfaction; IIEF = International Index of Erectile Function; ^*∗*^*p* < 0.05 versus “Yes” group.

**Table 5:** Logistic regression models assessing clinical, hormonal, and genetic factors associated with high values of sexual parameters in eugonadal subjects.

Dependent variable	Variables entered into models	OR (95% CI)	*p*	Model significance
EF	Age	0.784 (0.520–1.183)	NS	*p* < 0.001
	Hypertension (yes)	0.041 (0.004–0.410)	0.007	
	CAG triplets	0.568 (0.386–0.835)	0.004	

OF	Age	0.553 (0.311–0.982)	0.043	*p* < 0.001
	Hypertension (yes)	0.005 (0.0001–0.190)	0.004	
	CAG triplets	0.649 (0.427–0.988)	0.044	

SD	Age	0.798 (0.467–1.363)	NS	*p* = 0.001
	Hypertension (yes)	32.337 (0.355–2942.2)	NS	
	CAG triplets	0.108 (0.012–0.989)	0.049	

IS	Age	0.913 (0.586–1.423)	NS	*p* < 0.001
	Hypertension (yes)	0.033 (0.002–0.490)	0.013	
	CAG triplets	0.684 (0.461–1.016)	NS	

OS	Age	0.553 (0.311–0.982)	0.043	*p* < 0.001
	Hypertension (yes)	0.005 (0.0001–0.190)	0.004	
	CAG triplets	0.649 (0.427–0.988)	0.044	

Total IIEF	Age	0.784 (0.520–1.183)	NS	*p* < 0.001
	Hypertension (yes)	0.041 (0.004–0.410)	0.007	
	CAG triplets	0.568 (0.386–0.835)	0.004	

EF = erectile function; OF = orgasmic function; SD = sexual desire; IS = intercourse satisfaction; OS = overall satisfaction; IIEF = International Index of Erectile Function; NS, not significant; OR, odds ratio; CI, confidence interval.
